# Establishment and characterization of a metastasis model of human gastric cancer in nude mice

**DOI:** 10.1186/s12885-016-2101-z

**Published:** 2016-02-03

**Authors:** Kesheng Li, Huifen Du, Xiaowen Lian, Dandan Chai, Xinwen Li, Rong Yang, Chunya Wang

**Affiliations:** Department of Medicine Biotechnology, Medicine and Science Research Institute of Gansu province, Lanzhou, China; Department of Surgery, Tumor Hospital of Gansu province, Lanzhou, China; Department of pathology, Tumor Hospital of Gansu province, Lanzhou, China

**Keywords:** Characterization, Establishment, Gastric cancer, Metastasis, Mouse models

## Abstract

**Background:**

A mouse model of metastasis of human gastric cancer is one of the most important tools for studying the biological mechanisms underlying human gastric cancer metastasis. In this paper, we established a mouse model of metastatic human gastric cancer in nude mice that has a higher rate of tumor formation and metastasis than existing models.

**Methods:**

To generate the mouse model of metastatic human gastric cancer, fresh tumor tissues from patients that have undergone surgery for gastric cancer were subcutaneously implanted in the right and left groins of nude mice. When the implanted tissue grew to 1 cubic centimeter, the mice were killed, and the tumor tissues were examined and resected. The tumor tissues were implanted into nude mice and subjected to pathological examination, immunohistochemical staining, and real-time PCR for cytokeratin 8/18 (CK8/18), E-cadherin, vascular cell adhesion molecule-1 (VCAM-1) and intercellular adhesion molecule-1 (ICAM-1). The mice were also analyzed for metastasis in their peritoneum, abdominal cavity, and internal organs by histopathological examination. Tissues collected from these organs were examined for pathology.

**Results:**

After ten generations of implantation, all mice developed tumor growth at the implanted position, 94 % of the mice developed metastasis to the retroperitoneum and viscera. The implanted and metastatic tumor maintained the same histological features across all generations, and metastasis was observed in the esophagus, stomach, spleen, liver, kidney, adrenal, intestine, and pancreas. These metastatic tumors revealed no detectable expression of CK8/18, E-cadherin, VCAM-1, and ICAM-1.

**Conclusions:**

This model will serve as valuable tool for understanding the metastatic process of human gastric cancer.

## Background

Gastric cancer is the fourth most common malignancy and the second leading cause of cancer deaths only to lung cancer in the world [1]. Although the prognosis of patients with early gastric cancer has been prolonged distinctly by current methods of diagnosis and treatment, the 5-year survival rate after diagnosis of gastric cancer patients with all stages is <50 % [2]. Metastasis accounts in part for the high mortality from gastric cancer. The proportion of patients with gastric cancer dying from peritoneum metastasis is approximately 50 % [3]. Therefore, metastasis has become a focus of many gastric cancer studies. Metastasis is a very complex process, involving multiple consecutive steps [4]. Genes associated with cell adhesion, motility, proliferation, survival, metabolism, and signal transduction play an important role in cancer metastasis [5–8]. How these proteins work collectively to promote metastasis remains poorly understood.

A mouse model of metastatic gastric cancer is an extremely valuable tool in understanding the metastatic process. The first human carcinoma model in nude mice was established in 1969 by Rygaard and Povlsen through hypodermical transplantation of human colon cancer tissue [9]. Although the transplanted tumor retained its malignant characteristics, it lost its metastatic potential, and the original structure and behavior of the tumor changed [10]. A metastatic model of human colon cancer was first constructed by Morikawa in 1988 using human colon cancer cells subserously implanted into cecum [11]. This model showed orthotopic tumor growth and liver metastasis. Furukawa further modified this model in 1993 by surgically stitching human gastric cancer tissue into the tunica serosa gastria of nude mice [12]. This model developed tumors robustly and showed a very high rate of metastasis to the liver. Since disruption of the adhesion of the tumor tissue alters its biological and malignant behavior, the mouse models described retained the integrity of the tumors allowing for a “patient-like-model” [13, 14]. Hereafter, many mouse models of metastatic human gastric cancer have been generated by orthotopic transplantation of gastric cancer tissue [15–18].

The mouse models of metastatic human gastric cancer reported so far pose multiple challenges; the orthotic implantation into nude mice required surgery, and the tumor tissues implanted were derived from human gastric cancer cell line instead of patients. As a result, the procedure is lengthy and could cause heavy bleeding and death in mice. Moreover, although the rate of orthotopic tumor formation is nearly 80–100 %, the rate of metastasis not as high; the liver tumor metastatic rates were at 45–60 % [16, 17] and that with the peritoneum at a merely 40 % [18]. Thus, establishment of these mouse models could benefit from improved methods that would make transplantation easier and result in a more robust metastasis. In this report, we described a mouse model of metastatic human stomach cancer that addresses the issues from previous mouse models. We established our mouse model of metastatic human stomach cancer through subcutaneous implantation of tumor tissues derived surgically directly from patients with gastric cancer. Compared to other mouse models described previously, this mouse model forms tumors at a high rate and more importantly, shows robust metastasis.

## Methods

### Ethics statement

All the protocols involving the use of experimental animals and tumor tissues from patients with gastric cancer in this study were approved by the Ethics Committee of Medicine and Science Research Institute of Gansu Province (laboratory animals science group and clinical trial group, reference number: P201108150024), the approved programs included the collection, processing and implantation of tumor tissues from patients with gastric cancer , and the resection, storage and examination of tumor tissues from nude mice. All study participants provided informed consent to participate in the study.

### Animals and clinical tumor tissues

BALB/C nude mice at 4–6 weeks of age and 16–18 g in weight, both male and female, were provided by Shanghai Tumor Institute and reared in specific-pathogen-free (SPF) condition. Tumor tissues were obtained from patients with gastric cancer who underwent surgery in the Gansu Tumor Hospital. The fresh tumor tissues were implanted immediately after resection. Clinical data of the patients are listed in Table 1.

**Table 1:** Clinical data

Sample^a^	Histopathologic classification	Clinical stages	Rate of lymph node metastasis
1st	poorly differentiated adenocarcinoma	PT4aN3a M0 IIIc	7/30
2nd	moderately differentiated adenocarcinoma	PT4aN0M0 IIb	0/12
3rd	ulcerative type moderately differentiated adenocarcinoma	PT4aN0M0 IIb	0/30
4th	poorly differentiated adenocarcinoma	PT4aN3a M0 IIIc	16/17

^a^1st and 4th gastric cancer tissue were poorly differentiated adenocarcinoma and have lymph node metastasis. 2nd and 3rd were moderately differentiated adenocarcinoma and have no lymph node metastasis

### Subcutaneous implantation of fresh tumor tissues into nude mice

The fresh tumor tissues resected from patients with gastric cancer were cut to 1 cubic millimeter pieces which were diluted with DMEM medium, and then subcutaneously implanted into the right and left armpits and groins of nude mice with 16-gague needle under aseptic condition. Each sample was implanted to four mice, 5–6 pieces (0.8 mL) per mouse. The nude mice were subsequently reared in SPF condition, and the tumor growth on nude mice was examined daily. Once the tumor on the nude mice has grown to 1 cubic centimeter, the mouse was killed by cervical dislocation and the tumor tissues were examined and resected in aseptic condition. The tumor tissue from each mice was separated into three parts - one was used for another round of implantation into nude mice, the other was fixed in 10 % formaldehyde for pathological examination and immunohistochemical (IHC) staining for CK8/18, E-cadherin, VCAM-1, and ICAM-1, the third was stored in liquid nitrogen and in −80 °C for real-time PCR analysis of CK8/18, E-cadherin, VCAM-1, and ICAM-1. The mice were dissected and examined for tumor metastasis in their peritoneum, abdominal cavity, liver, spleen, stomach, intestines, kidneys, lung, and brain. Collected tissues were fixed in 10 % formaldehyde for pathological examination.

### Establishment and characterization of mouse model of metastatic human gastric cancer

The excised tumor tissue was subcutaneously implanted to the right and left groins of 5 nude mice under aseptic condition using 5–6 pieces (0.8 mL) per mouse. The cutting and diluting of tumor tissue, growth examination and resection of the tumor in the nude mice, storage and examination of the implanted tumor tissues, metastatic tumor tissues, and mouse bodies were processed as described above. Implanted tumor tissues were passaged for ten generations.

### Examining effect of the site of implantation on the rate of metastasis

As described, the implanted human gastric cancer tissue from nude mice was subcutaneously implanted to three groups of nude mice at different sites under aseptic condition. An average of 5–6 pieces were implanted into mice, with one group receiving the tissues at the right and left groins, the other group at the right and left armpits, and the third at two sites in the back. As mentioned above, growth examination and resection of the tumor in the nude mice were processed. Further analyses included examination of tumor growth at different sites and metastasis in the peritoneum and abdominal cavity.

### Implantation and metastasis of previously frozen and passaged human gastric cancer tissue in nude mice

The implanted human gastric cancer tissues passaged from fourth and eighth generation by implantation into nude mice were stored in liquid nitrogen and subcutaneously implanted into nude mice at the right and left groins as described above. Further analyses included examination of tumor growth at different sites and metastasis in the peritoneum and abdominal cavity.

### Pathological examination of the implanted and metastatic human gastric tissues from nude mice

The implanted and metastatic human gastric cancer tissues from nude mice were fixed in 10 % formaldehyde, embedded in paraffin, cut into sections, stained in Hematoxylin-eosin staining (HE). The slides were evaluated using an Olympus BX50 light microscope, and image acquisition was performed by Mias pathological workstation 4.0 system.

### IHC staining

The expression levels of E-cadherin, VCAM-1, ICAM-1, and CK8/18 were examined by immunohistochemistry in the implanted and metastatic tumor tissues from nude mice and in the surgical specimens used for implantation. Sections used for staining were obtained from the surgical specimens, the implanted and metastatic tumor tissues, and the tissues that contain metastatic tumors. Reagents used for staining were SP-9000 Histostain™-plus Kits, 3-3′-Diaminobenzidine tetrahydrochloride (DAB) Kits, primary mouse monoclonal antibodies against E-cadherin (1:200 dilution), ICAM-1 (1:500 dilution), and primary rabbit polyclonal antibody against VCAM-1 (1:500 dilution) (Beijing Zhongshan Golden Bridge Biotechnology Co Ltd., Beijing, China). The IHC staining slides were independently assessed by two pathologists, and any difference in the decision outcome was resolved by consensus. Staining intensity was assessed as negative, weak, moderate, or strong. The light microscope and image acquisition software were the same as above.

### Total RNA extraction and real-time PCR

Total RNA was extracted by Trizol (Sheng gong Biotechnology, Shanghai, China) from the implanted and metastatic tumor tissues that grew in nude mice and from the surgical specimens used for implantation, following manufacturer’s instructions. The cDNA was synthesized by reverse transcriptase (Sheng gong Biotechnology), according to the manufacturer’s recommendations. The SYBR premix Ex TaqTM (TaKaRa Biotechnology, Dalian, China) was used for the real-time PCR. The 20-μl reaction contained 10 μl SYBR premix Ex TaqTM, 1 μl DNA template, 0.4 μl each primer, and 8.2 μl dH_2_0. The PCR cycling condition was: 37 °C for 5 min, 95 °C for 30 s, and 40 cycles of 95 °C for 5 s to 60 °C for 30 s. The β-actin mRNA was used as internal control, and the reaction mix without the template DNA was used as negative control. All of the samples were measured 3 times independently, and the quantitative PCR data were analyzed using the comparative CT method. Briefly, the difference in cycle threshold, ∆CT, was determined as the difference between the tested gene and human β-actin. We then obtained ∆∆CT by finding the difference between the two groups. The fold change was calculated as 2 ^-∆∆CT^. The primers are listed in Table 2.

**Table 2:** Primers used in the real-time PCR

Gene	Forward primer	Reverse primer
β-actin	5'-TGGCACCCAGCA	5'-CTAAGTCATAGT
	CAATGAA-3'	CCGCCTAGAAGCA-3'
E-cadherin	5'-GAGTGCCAACTG	5'-AGTCACCCACCT
	GACCATTCAGTA-3'	CTAAGGCCATC-3'
ICAM-1	5'-TGTATGAACTGA	5'-CACCTGGCAGCG
	GCAATGTGCAAGA-3'	TAGGGTAA-3'
VCAM-1	5'-GGCGCCTATACC	5'-AGAGCACGAGAA
	ATCCGAAA-3'	GCTCAGGAGAA-3'

## Results

### Tumor formation and metastasis

**Fig. 1:**
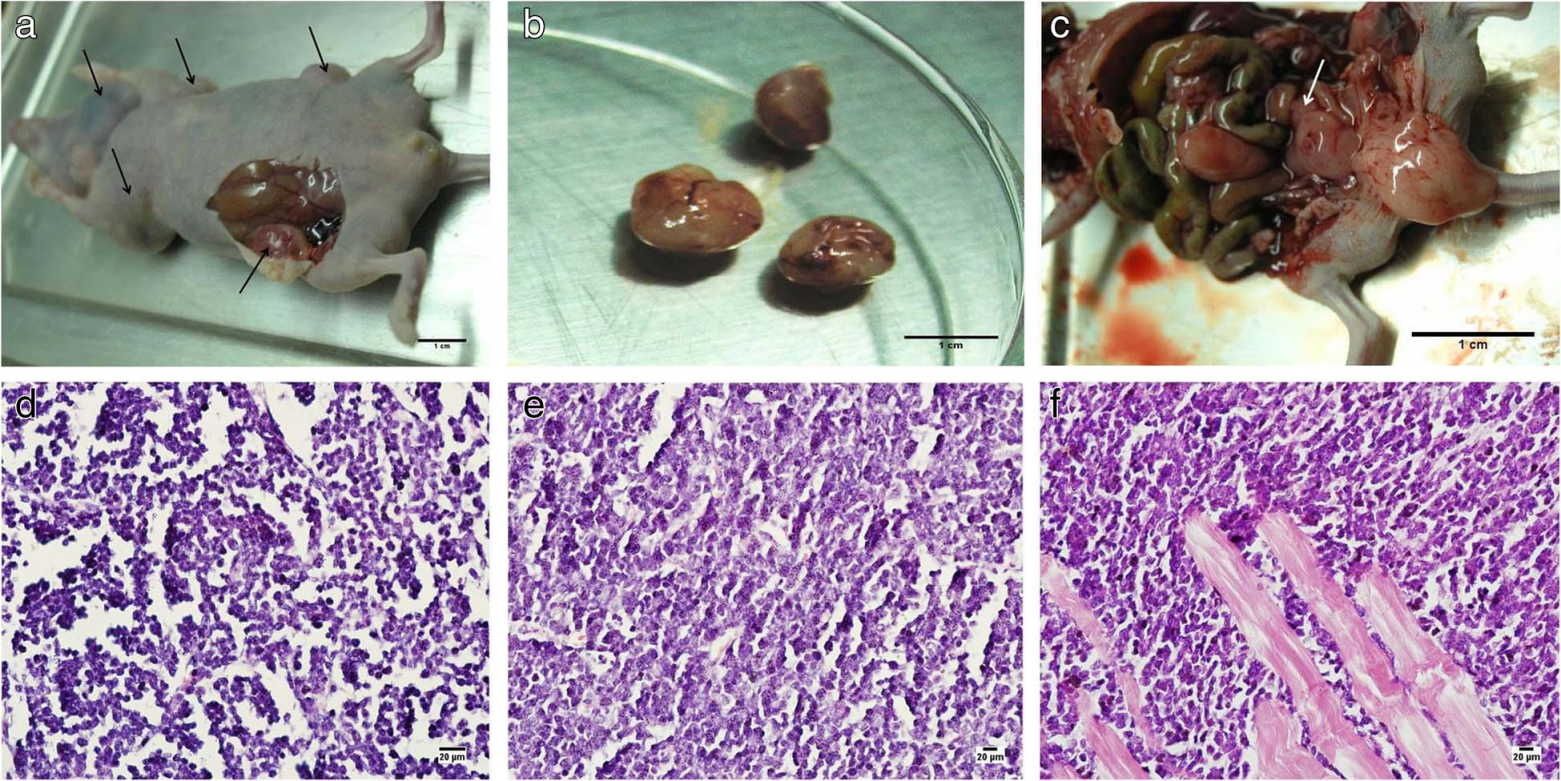
Metastatic tumor growth from the implanted gastric cancer tissue obtained surgically (X 400). **a**, **b**: Implanted cancer tissue grew to ~1 cubic centimeter and displayed an intact envelope and hard texture; **c**: Tumor metastasized into the mouse retroperitoneum; **d**, **e**: The implanted tissue and metastatic tumors consisted of poorly differentiated carcinoma cells and a few mesenchyma cells and blood vessels, with some resemblance to glandular cavity. The cancer cells showed dark-stained nuclei and scant cytoplasm and lacked the normal proportion between nucleus and cytoplasm; **f**: Implanted tumor showing tissue infiltration

Among the four mice implanted with the 1st surgical specimens, only one developed tumor at the site of implantation by 76 days (Fig. 1a). Twenty-five days later, the mouse was killed by cervical dislocation and analyzed for tumors. Tumor tissue at an average of 1 cubic centimeter in size, displayed an intact envelope and hard texture (Fig. 1b). Metastasis in the retroperitoneum was found by visual inspection (Fig. 1c). No metastasis was detected in its peritoneum, abdominal cavity, liver, spleen, stomach, intestines, kidneys, lung, and brain.

Pathological analysis revealed that the implanted and metastatic tumor tissues consisted of poorly differentiated carcinoma cells, and only a little of mesenchyma and blood vessel. These tissues appear diffused, lacked structure, and resemble glandular lumen. Moreover, the cells displayed dark-stained nuclei, scant cytoplasm, and misproportioned nuclei and cytoplasm (Fig. 1d, e, f). Similar results were obtained in a parallel study involving implantation of the tumor tissue into 4 mice; only one mouse developed tumor (average size: 1.5 cubic centimeter) 26 days after implantation. No metastasis was observed in its peritoneum, abdominal cavity, liver, spleen, stomach, intestines, kidneys, lung, and brain. The other mice implanted with the 2nd and 4th surgical specimens showed no tumor growth.

### Stability of the implanted tumor following passage into multiple generations

**Fig. 2:**
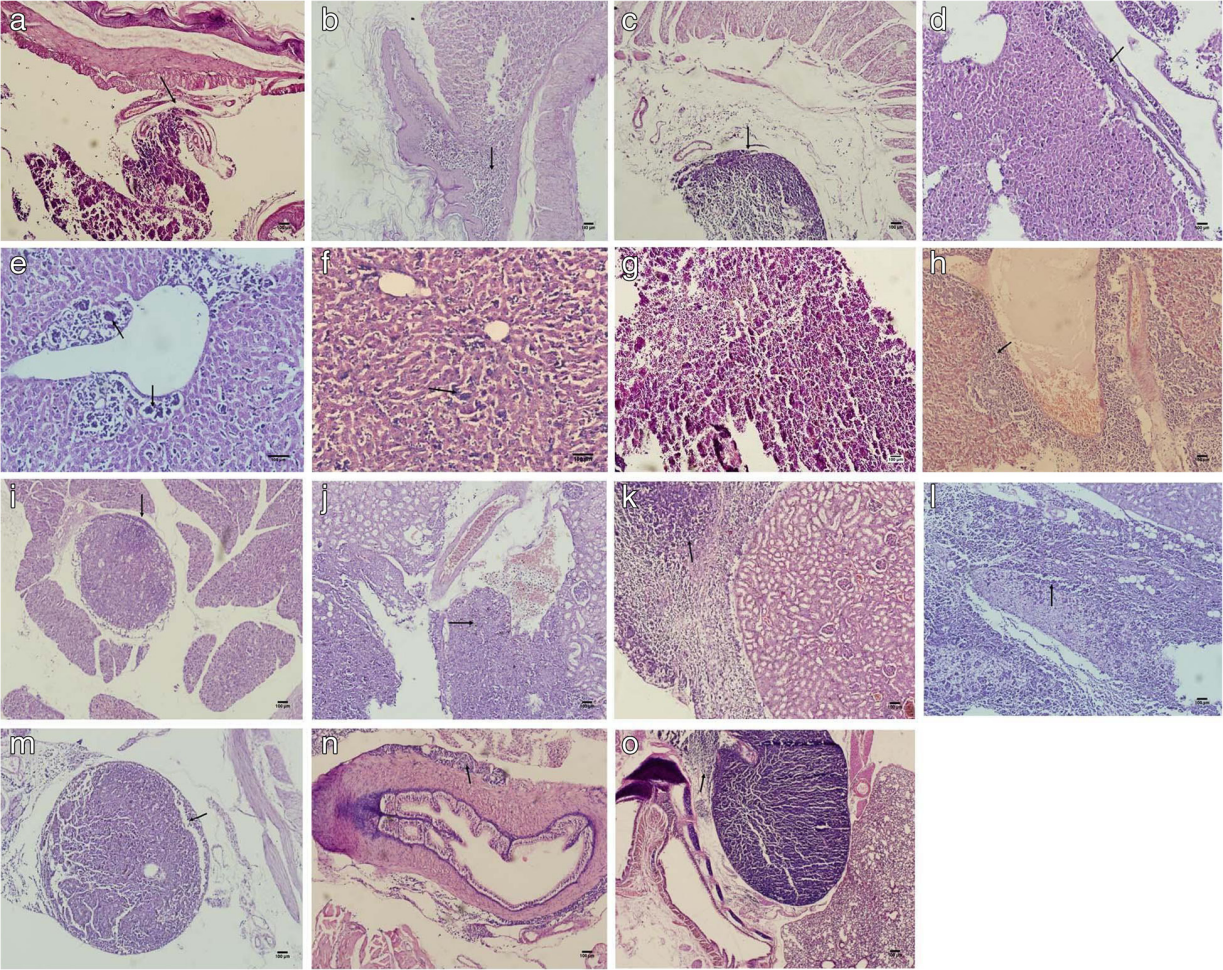
Pathological examination of the tumor that metastasized to the viscera (X 100). Micro-metastasis was observed in the lymph nodes around the esophagus (**a**), below the gastric mucosa (**b**), and in other areas such as tunica serosa gastria (**c**), parenchyma under hepatic capsule (**d**), liver portal area (**e**), sinus hepaticus (**f**), spleen (**g**), venae centrals hepatic (**h**), pancreas (**i**), renal hilum (**j**), renal parenchyma (**k**), adrenal gland (**l**), intestine serosa (**m**), spermaduct (**n**), and lung (**o**)

The tumor that developed from the 1st surgical specimen was passaged for ten generations. The rate of tumor growth was 100 % and that of metastasis in retroperitoneum and viscera was 80–100 % (average 94 %), regardless whether the primary tissue was used fresh or frozen (Table 3). The viscera metastasis was observed in the lymph nodes around esophagus, below gastric mucosa, tunica serosa gastria, spleen, liver portal area, central venae and sinus hepaticus, liver parenchyma, liver capsule, renal hilum, kidney parenchyma, adrenal gland, intestine serosa, pancreas, and spermaduct (Fig. 2). The generation time is 16 days.

**Table 3:** Stability and rate of metastasis of tumors implanted at different positions^a^

Variable	No	Growth in implanted position (%)	Metastasis retroperitoneum (%)	Viscera (%)	Generation time (days)
Passage number					
1st	5	100 (5/5)	100 (5/5)	80 (4/5)	20
2nd	5	100 (5/5)	80 (4/5)	100 (5/5)	14
3rd	5	100 (5/5)	100 (5/5)	80 (4/5)	14
4th	5	100 (5/5)	80 (4/5)	100 (5/5)	15
5th	5	100 (5/5)	100 (5/5)	80 (4/5)	14
6th	5	100 (5/5)	80 (4/5)	100 (5/5)	13
7th	5	100 (5/5)	100 (5/5)	100 (5/5)	17
8th	5	100 (5/5)	100 (5/5)	100 (5/5)	18
9th	5	100 (5/5)	100 (5/5)	100 (5/5)	17
10th	5	100 (5/5)	100 (5/5)	100 (5/5)	18
Stored in liquid nitrogen					
4th	5	100 (5/5)	100 (5/5)	100 (5/5)	17
8th	5	100 (5/5)	100 (5/5)	100 (5/5)	18
Implantation in different position					
Groins	50	100 (50/50)	94 (47/50)	94 (47/50)	16
Back	10	100 (10/10)	30 (3/10)	10 (1/10)	20
Armpits	10	100 (10/10)	0 (0/10)	30 (3/10)	14

^a^ After ten generations of implantation, all mice developed tumor growth at the implanted position, and 94 % of mice developed metastasis to the retroperitoneum and viscera, regardless whether the tumor source was fresh or frozen. The average time of bearing tumor is 16 days. The groin of mice is best Implantation position, resulting in 94 % retroperitoneum and viscera metastasis

### The rate of metastasis of the tumor implanted into different positions

Implantation into different positions affected the rate of metastasis but not the rate of tumor growth. Implantation into the groin resulted in 94 % retroperitoneum and viscera metastasis; implantation into the back resulted in 30 % retroperitoneum metastasis and 10 % viscera metastasis; implantation into armpits resulted in no retroperitoneum metastasis and 20 % viscera metastasis. The generation time was: 16 days for tumors implanted in the groins, 20 days for those implanted in the back, and 14 days for those implanted in the armpits (Table 3). The metastatic viscera included liver (50 %), kidney (44 %), intestine (28 %), esophagus (12 %), pancreas (12 %), stomach (6 %), spleen (6 %), and spermaduct (6 %) (Table 4).

**Table 4:** Metastasis into the viscera^a^

Implanted position	No	viscera metastasis (%)	Liver (%)	Kidney (%)	Intestine (%)	Esophagus (%)	Pancreas (%)	Stomach (%)	Spleen (%)	Spermaduct (%)
Groins	50	94 (47/50)	50 (25/50)	44 (22/50)	28 (14/50)	12 (6/50)	12 (6/50)	6 (3/50)	6 (3/50)	6 (3/50)
Back	10	10 (1/10)	0	0	0	0	0	10 (1/10)	10 (1/10)	0
Armpits	10	30 (3/10)	20 (2/10)	0	0	10 (1/10)	0	0	0	0

^a^ Liver and kidney were the viscera with highest rate of metastasis (44–50 %), and the stomach, spleen and spermaduct were the lowest (6 %)

### Characterization of the implanted and metastatic tumor

**Fig. 3:**
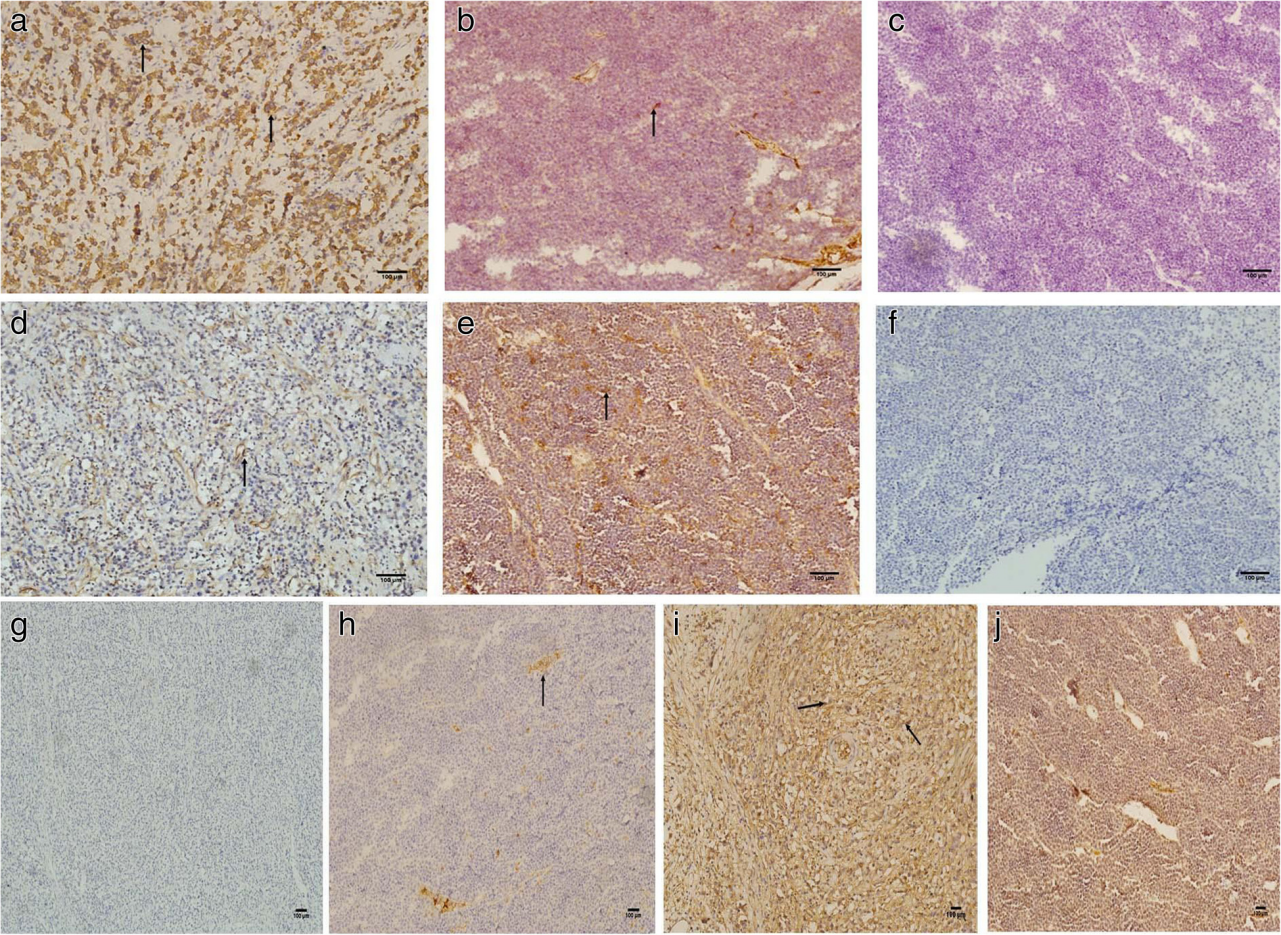
IHC analysis of the expression of E-cadherin, VCAM-1, ICAM-1 and CK8/18 (X 200). CK8/18 expression was detected in the surgical specimen used for implantation (**a**) and in the primary implanted tumor tissues (**b**), but not in the F1 generation implanted tumor tissues (**c**), VCAM-1 was expressed in the surgical specimen (**d**), and in the primary implanted tumor tissues (**e**), but not in the F2 generation implanted tumor tissues (**f**). E-cadherin expression was not detectable in the surgical specimen (**g**) and in the primary implanted tumor tissues (**h**). ICAM-1 was expressed in the surgical specimen (**i**), but not in the primary implanted tumor tissues (**j**)

The IHC and real-time PCR results revealed that ICAM-1, VCAM-1, and CK8/18, but not E-cadherin, were predominantly expressed at surgery and in the implanted tumor of primary and first generation (Fig. 3). As shown in Table 5, the primary and first generation of the tumor showed positive staining for VCAM-1 and CK8/18, but the subsequent generations showed weak staining for these proteins: VCAM-1 staining was scored as moderately positive (++) in the primary, weak signal (+) in the first generation, and CK8/18 staining was scored as weak signal (+) in the first generation. Tumors at all stages showed negative staining for E-cadherin, whereas metastatic tumor at all generations showed negative staining for E-cadherin, ICAM-1, VCAM-1, and CK8/18. As for the transcripts, we detected VCAM-1 mRNA in the primary and first generation implanted tumor but not at the metastatic stage. E-cadherin and ICAM-1 transcripts were not detected in all generations of implanted and metastatic tumors.

**Table 5:** Expression of E-cadherin, ICAM-1, VCAM-1 and CK8/18 in the tumors at surgery, upon implantation and during metastasis

Variable	No	Protein expression^a^				mRNA expression^a^		
		E-cadherin	ICAM-1	VCAM-1	CK8/18	E-cadherin	ICAM-1	VCAM-1
surgical specimen	1	-	+++	+++	+++			
Primary	1							
Implanted tumor		-	-	++	+	0	0	0.0927
Metastatic tumor		-	-	-	-	0	0	0
1st	5							
Implanted tumor		− (0/5)	− (0/5)	+ (5/5)	− (0/5)	0	0	0.1997
Metastatic tumor		− (0/5)	− (0/5)	− (5/5)	− (0/5)	0	0	0
2nd ~ 10th	45							
Implanted tumor		− (0/45)	− (0/45)	− (0/45)	− (0/45)	0	0	0
Metastatic tumor		− (0/42)	− (0/42)	− (0/42)	− (0/42)	0	0	0

^a^Molecular analysis of the implanted and metastatic tumors revealed no detectable expression of CK8/18, E-cadherin, VCAM-1 and ICAM-1, except positive staining for VCAM-1 in the implanted tumor tissues of the first generation

## Discussion

Cancer is characterized by proliferation, invasion, and metastasis. More than 90 % of mortality from cancer is due to metastasis thereby prompting intense research [19]. Metastasis is a complicated and poorly understood process involving proteins with functions in cell adhesion, ECM degradation, and motility [19–21]. Numerous studies on gastric cancer metastasis have been reported [22–26]. However, most of these studies were conducted in vitro, failing to mimic the metastatic process that occurs in vivo. This suggests a need for an animal model of cancer metastasis that has a robust and consistent phenotype. In the present study, a model of metastatic human gastric cancer was established by hypodermic inoculation in nude mice with cancer tissues obtained surgically from patients with gastric cancer. All mice developed tumor growth at the implanted position and retroperitoneum metastasis, and 94 % of mice developed metastasis to the viscera, regardless whether the tumor source was fresh or frozen. The implanted and metastatic tumor maintained the same features across all generations, and the viscera metastasis was observed in lymph nodes around the esophagus, below the gastric mucosa, tunica serosa gastria, spleen, liver portal area, central venae and sinus hepaticus, liver parenchyma, liver capsule, renal hilum, kidney parenchyma, adrenal gland, intestine serosa, and pancreas. Metastasis was robust in this mouse model. The retroperitoneum metastasis possibly resulted from the dissociation of tumor cells from the implanted tumor, introduction into the inguinal glands, and transport to the retroperitoneum. This may account for the tumor metastasizing into liver portal area, central venae, and sinus hepaticus, as well as into tunica serosa gastria, renal hilum, adrenal gland, and intestine serosa. Metastasis could also have occurred through the lymph nodes; tumors were observed in the lymph nodes around esophagus, below gastric mucosa, spleen, pancreas, and kidney parenchyma.

The occurrence of metastasis appears to be dependent on the site of implantation subcutaneously: implantation into the groin resulted in 100 % retroperitoneal metastasis and 94 % viscera metastasis; implantation into the back resulted in 30 % retroperitoneum metastasis, and 10 % viscera metastasis; implantation into armpits resulted in no retroperitoneum metastasis and 20 % viscera metastasis. This observation is consistent with metastasis associated with tumor growth microenvironment including blood vessel and lymph distribution. Indeed, the mouse groin has more blood vessels and lymph networks that flow into abdominal cavity and viscera than the back. Although the armpits have rich blood vessels and lymphatic networks, the direction of the vena is anterograde, and most of lymph connect with lung, trachea and pleura, locations where gastric cancer seldom gets translocated. Therefore, the simple method of subcutaneous implantation of cancer cells into the groins of nude mice efficiently results in a model of metastatic human gastric cancer. This model has a higher viscera metastasis rate than that reported in the literature [13–18] and could easily be applied to other types of human cancer.

Tumor invasion with subsequent metastases is the major cause of morbidity and mortality in patients with cancer. Cancer metastasis is a complex process in which tumor cells separate from the primary tumor mass, migrate through the vascular system, extravasate into other tissues and grow into new tumors [27–30]. Among these diverse processes, an alteration in the adhesive properties of the primary tumor cells is a critical factor for tumor progression [28]. It has been revealed that cell adhesion is responsible for tumor progression, involving molecules that play a role in cell-cell adhesion and cell-matrix adhesion [31–34]. Cell adhesion plays an important role in the two different stages of the tumor metastatic process - the detachment from the primary tumor and its adhesion to the circulatory system [27]. Therefore, cell adhesion molecules play a critical role in the invasion and metastasis of a variety of human tumors.

E-cadherin plays an important role in cell-cell adhesion in epithelial tissues [35]. Besides its role in normal cells, this cell adhesion molecule can play a major role in malignant cell transformation, tumor development, and progression. The loss of tumor tissue integrity can lead to local invasion [36]. Therefore, loss of function of E-cadherin in tumor tissues correlates with invasiveness and metastasis of tumors [37]. Studies have shown that aberrant E-cadherin expression is associated with the acquisition of invasiveness and more advanced tumor stage for gastric cancer [38–40].

ICAM-1 and VCAM-1 are very important cell adhesion molecules belonging to the immunoglobulin super family. The ICAM-1 functions in cell-cell and ECM adhesion, including physiological polymorphonuclear (PMN) tight adhesion and trans endothelial migration via the leukocyte integrins lymphocyte functionassociated antigen-1 (LFA-1) (CD11a/CD18) and macrophage-1 antigen (MAC-1) (CD11b/CD18) [41]. The VCAM-I mediates cellular adhesion via integrin [42]. ICAM-1 plays an important role in cell-cell and cell-ECM interactions, especially tumor invasion and cytotoxicity of lymphocytes. Studies have shown that the positive expression rate of ICAM-1 was related with lymph node metastasis and depth of tumor invasion, and the VCAM-1 expression positive gastric cancers were more invasive and were associated with more lymph node metastases than VCAM-1 expression negative ones [43–45]. Cytokeratin appear on all epithelial cells, some non-epithelial cells, and most tumor cells. The cytokeratins, belonging to the intermediate filament (IF) protein family, are primary components of horn cells and maintains the organization of epithelial tissues. Studies have found that the cytokeratins are very highly conserved and important for tissue differentiation. At present, more than 20 different cytokeratins have been identified [46], of which CK 8, 18, and 19 are the most abundant in simple epithelial cells. In the present study, the IHC and RT-PCR results revealed that the expression of E-cadherin is negative, and that of ICAM-1, VCAM-1, and CK8/18 are positive in the surgical specimen used for implantation, consistent with past studies [38, 40, 43, 45]. Interestingly, E-cadherin, ICAM-1, VCAM-1 and CK8/18 are not expressed in the implanted and metastatic tumor tissues of nude mice, suggesting that the molecular and biological characteristics of the implanted and metastatic tumors are different from the original tissue obtained surgically. These differential characteristics may provide insights into the metastatic process.

## Conclusions

Tumor metastasis is a complicated multi-step process. Although numerous genes and factors have been associated with tumor metastasis, the exact molecular mechanisms underlying this process remains poorly understood. In present study, we have established a mouse model of metastatic human gastric cancer with a robust metastatic phenotype, which will be valuable in understanding the molecular mechanisms underlying this process.

## Abbreviations

CK8/18: Cytokeratin 8/18
DAB: Diaminobenzidine
HE: Hematoxylin-eosin staining
ICAM-1: Intercellular adhesion molecule-1
IF: Intermediate filament
IHC: Immunohistochemical
LFA-1: Lymphocyte function-associated antigen-1
MAC-1: Macrophage-1 antigen
PMN: Polymorphonuclear
SPF: Specific-pathogen-free
VCAM-1: Vascular cell adhesion molecule-1
